# Supplementary material for: Interaction network rewiring and species’ contributions to community-scale flexibility
Source: PNAS Nexus. 2024 Mar 5;3(3):pgae047. doi: 10.1093/pnasnexus/pgae047 (PMC10914369; doi:10.1093/pnasnexus/pgae047)
Supplement: pgae047_Supplementary_Data [file pgae047_supplementary_data.pdf]

Supporting Information for

Interaction network rewiring and species' contributions to community-scale flexibility

Hirokazu Toju[1,2,3]*, Sayaka S. Suzuki[1], and Yuki G. Baba[4]

[1]Center for Ecological Research, Kyoto University, Otsu, Shiga 520-2133, Japan

[2]Laboratory of Ecosystems and Coevolution, Graduate School of Biostudies, Kyoto University, Kyoto 606-8501, Japan

[2]Center for Living Systems Information Science (CeLiSIS), Graduate School of Biostudies, Kyoto University, Kyoto 606-8501, Japan

[4]Institute for Agro-Environmental Sciences, NARO, Kannondai 3-1-3, Tsukuba, Ibaraki 305-8604, Japan.

*Hirokazu Toju

Email: toju.hirokazu.4c@kyoto-u.ac.jp

This PDF file includes:

Figures S1 to S8

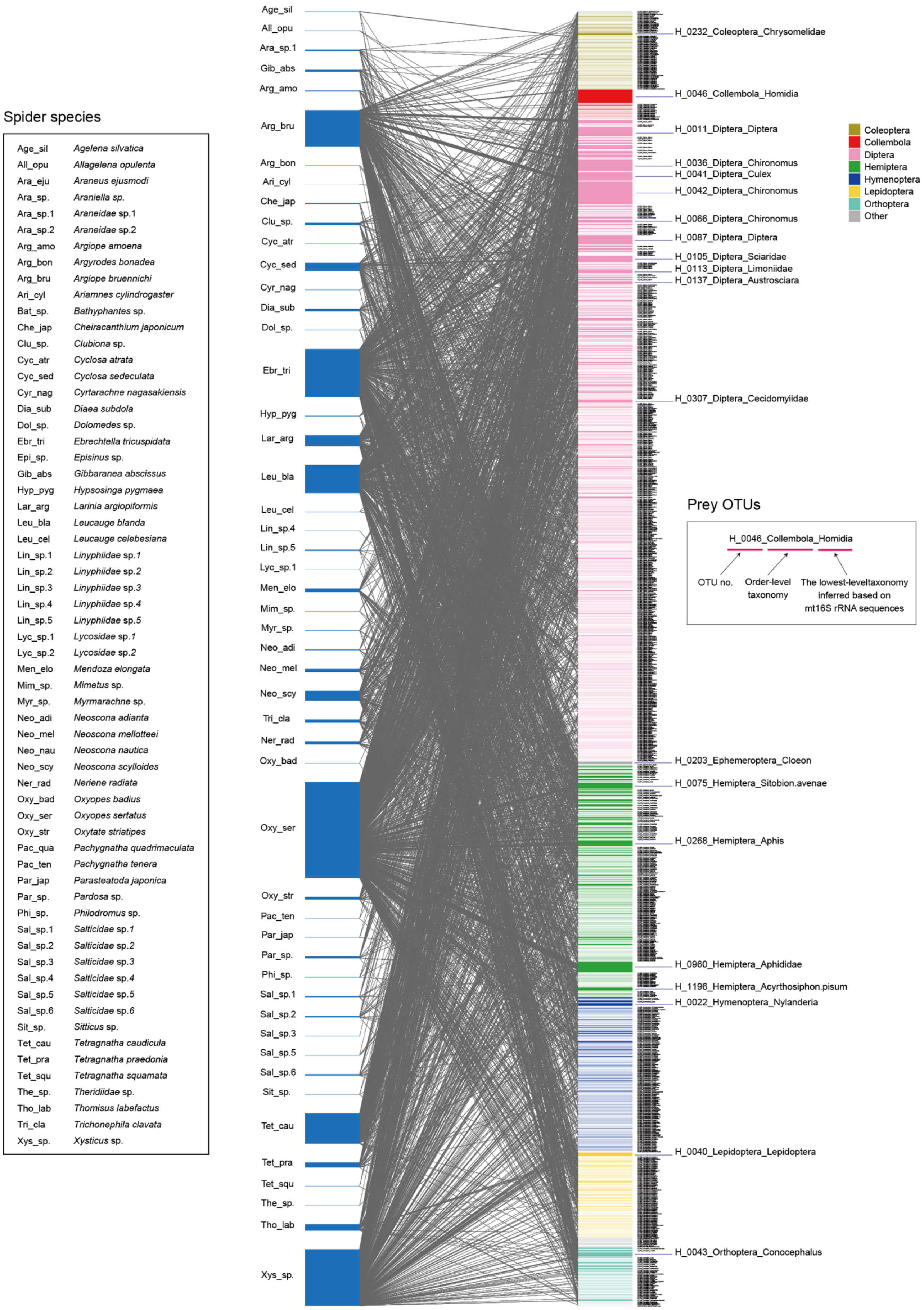
Spider species
Age_sil Agelena silvatica
All_opu Allagelena opulenta
Ara_eju Araneus ejusmodi
Ara_sp. Araniella sp.
Ara_sp.1 Araneidae sp.1
Ara_sp.2 Araneidae sp.2
Arg_amo Argiope amoena
Arg_bon Argyrodes bonadea
Arg_bru Argiope bruennichi
Ari_cyl Ariamnes cylindrogaster
Bat_sp. Bathyphantes sp.
Che_jap Cheiracanthium japonicum
Clu_sp. Clubiona sp.
Cyc_atr Cyclosa atrata
Cyc_sed Cyclosa sedeculata
Cyr_nag Cyrtarachne nagasakiensis
Dia_sub Diaea subdola
Dol_sp. Dolomedes sp.
Ebr_tri Ebrechtella tricuspidata
Epi_sp. Episinus sp.
Gib_abs Gibbaranea abscissus
Hyp_pyg Hypsosinga pygmaea
Lar_arg Larinia argiopiformis
Leu_bla Leucauge blanda
Leu_cel Leucauge celebesiana
Lin_sp.1 Linyphiidae sp.1
Lin_sp.2 Linyphiidae sp.2
Lin_sp.3 Linyphiidae sp.3
Lin_sp.4 Linyphiidae sp.4
Lin_sp.5 Linyphiidae sp.5
Lyc_sp.1 Lycosidae sp.1
Lyc_sp.2 Lycosidae sp.2
Men_elo Mendoza elongata
Mim_sp. Mimetus sp.
Myr_sp. Myrmarachne sp.
Neo_adi Neoscona adianta
Neo_mel Neoscona mellotteei
Neo_nau Neoscona nautica
Neo_scy Neoscona scylloides
Ner_rad Neriene radiata
Oxy_bad Oxyopes badius
Oxy_ser Oxyopes sertatus
Oxy_str Oxytate striatipes
Pac_qua Pachygnatha quadrimaculata
Pac_ten Pachygnatha tenera
Par_jap Parasteatoda japonica
Par_sp. Pardosa sp.
Phi_sp. Philodromus sp.
Sal_sp.1 Salticidae sp.1
Sal_sp.2 Salticidae sp.2
Sal_sp.3 Salticidae sp.3
Sal_sp.4 Salticidae sp.4
Sal_sp.5 Salticidae sp.5
Sal_sp.6 Salticidae sp.6
Sit_sp. Sitticus sp.
Tet_cau Tetragnatha caudicula
Tet_pra Tetragnatha praedonia
Tet_squ Tetragnatha squamata
The_sp. Theridiidae sp.
Tho_lab Thomisus labefactus
Tri_cla Trichonephila clavata
Xys_sp. Xysticus sp.
Age_sil
All_opu
Ara_sp.1
Gib_abs
Arg_amo
Arg_bru
Arg_bon
Ari_cyl
Che_jap
Clu_sp.
Cyc_atr
Cyc_sed
Cyr_nag
Dia_sub
Dol_sp.
Ebr_tri
Hyp_pyg
Lar_arg
Leu_bla
Leu_cel
Lin_sp.4
Lin_sp.5
Lyc_sp.1
Men_elo
Mim_sp.
Myr_sp.
Neo_adi
Neo_mel
Neo_scy
Tri_cla
Ner_rad
Oxy_bad
Oxy_ser
Oxy_str
Pac_ten
Par_jap
Par_sp.
Phi_sp.
Sal_sp.1
Sal_sp.2
Sal_sp.3
Sal_sp.5
Sal_sp.6
Sit_sp.
Tet_cau
Tet_pra
Tet_squ
The_sp.
Tho_lab
Xys_sp.
H_0232_Coleoptera_Chrysomelidae
H_0046_Collembola_Homidia
H_0011_Diptera_Diptera
H_0036_Diptera_Chironomus
H_0041_Diptera_Culex
H_0042_Diptera_Chironomus
H_0066_Diptera_Chironomus
H_0087_Diptera_Diptera
H_0105_Diptera_Sciaridae
H_0113_Diptera_Limoniidae
H_0137_Diptera_Austrosciara
H_0307_Diptera_Cecidomyiidae
H_0203_Ephemeroptera_Cloeon
H_0075_Hemiptera_Sitobion.avenae
H_0268_Hemiptera_Aphis
H_0960_Hemiptera_Aphididae
H_1196_Hemiptera_Acyrthosiphon.pisum
H_0022_Hymenoptera_Nylanderia
H_0040_Lepidoptera_Lepidoptera
H_0043_Orthoptera_Conocephalus
Coleoptera
Collembola
Diptera
Hemiptera
Hymenoptera
Lepidoptera
Orthoptera
Other
Prey OTUs
H_0046_Collembola_Homidia
OTU no.
Order-level taxonomy
The lowest-leveltaxonomy inferred based on mt16S rRNA sequences

Fig. S1. Details of the spider–prey meta-networks. All the spider–prey interactions observed from April to November are included in the meta-network. Spider species and prey Hexapoda OTUs are shown in the left and right, respectively.

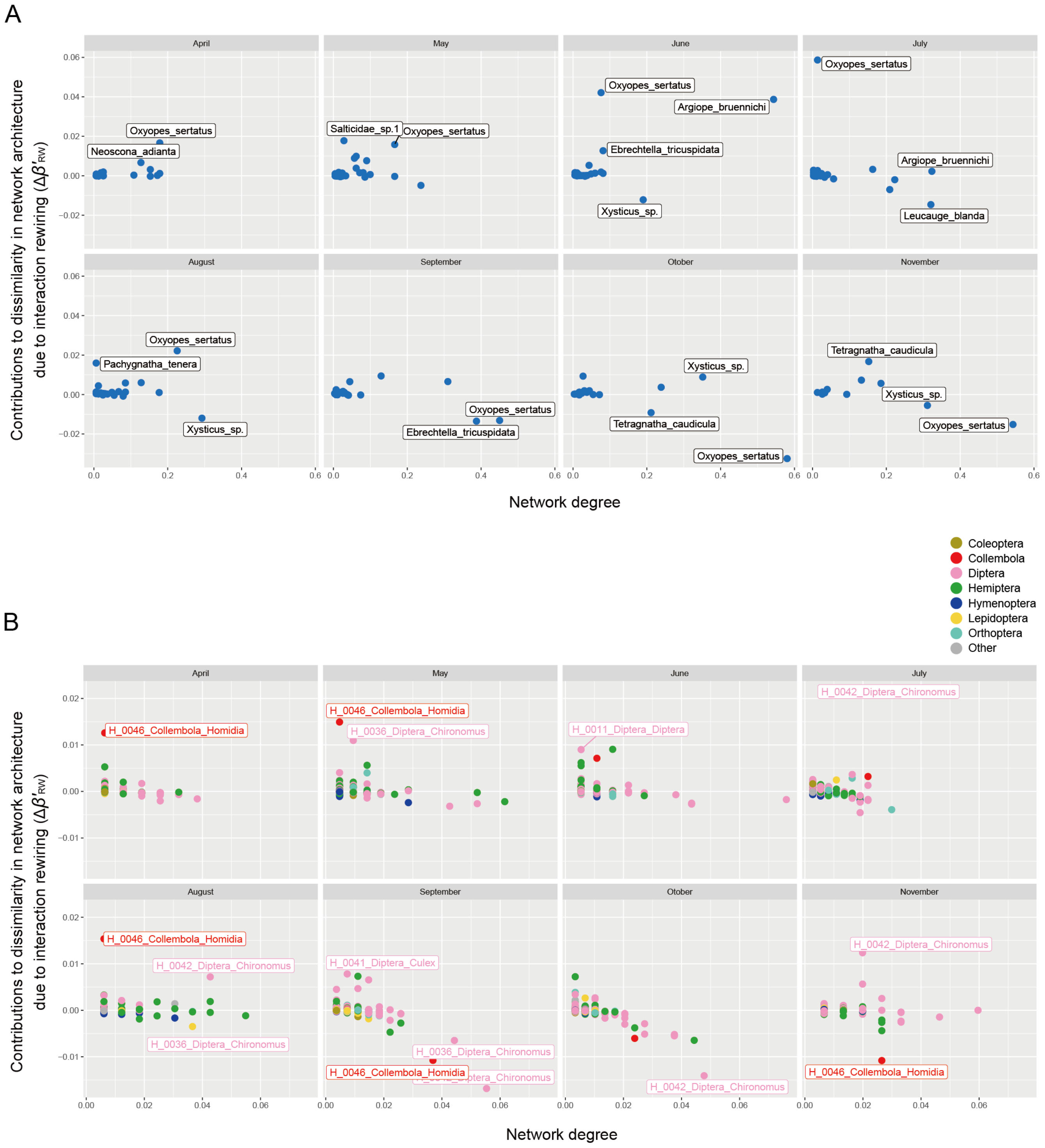

Fig. S2. Contributions of each species to dissimilarity in network architecture due to interaction rewiring. For each month, dissimilarity in network architecture due to interaction rewiring was calculated against the meta-network (Fig. 2A). Contributions of each spider species or prey OTUs to the dissimilarity levels ($\Delta\boldsymbol{\beta}'_{\mathbf{RW},i}$) were then evaluated as illustrated in Fig. 1B. The horizontal axis indicates network degree within the data of each month.

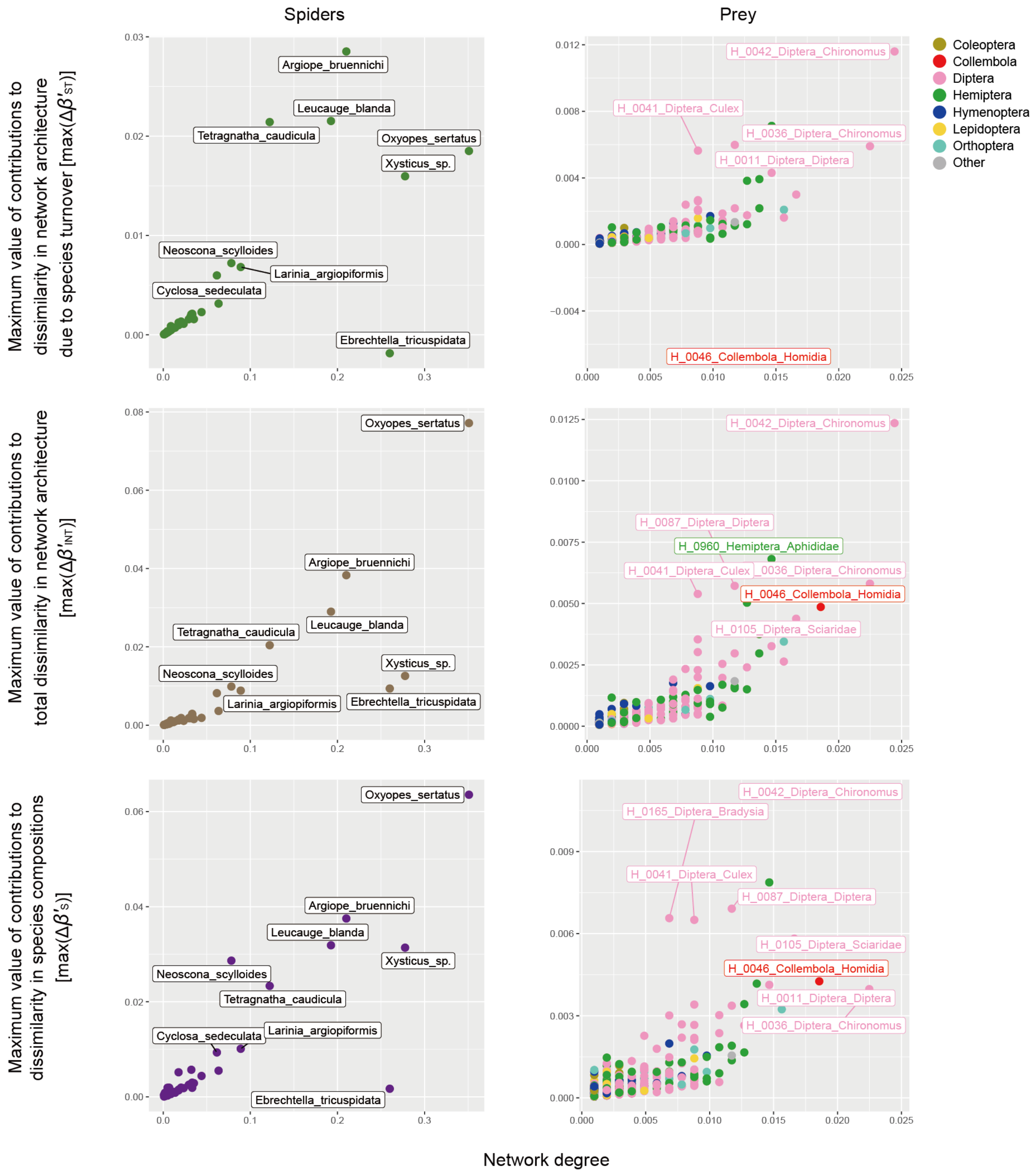

Fig. S3. Contributions of each species to various dissimilarity indices. In line with the maximum values of contributions to network rewiring effects [max($\Delta\boldsymbol{\beta}'_{\mathrm{RW},i}$)] shown in Figure S3, the maximum values of contributions to dissimilarity in network architecture due to species turnover [max($\Delta\boldsymbol{\beta}'_{\mathrm{ST},i}$)], the maximum values of contributions to total dissimilarity in network architecture [max($\Delta\boldsymbol{\beta}'_{\mathrm{INT},i}$)], and the maximum values of contributions to dissimilarity in species compositions [max($\Delta\boldsymbol{\beta}'_{\mathrm{S},i}$)] are shown respectively for spiders and prey. The horizontal axis indicates network degree within the meta-network (Fig. S1). The network degree was standardized by dividing the number of links by *N* -1, where *N* was the total number of vertices within the network.

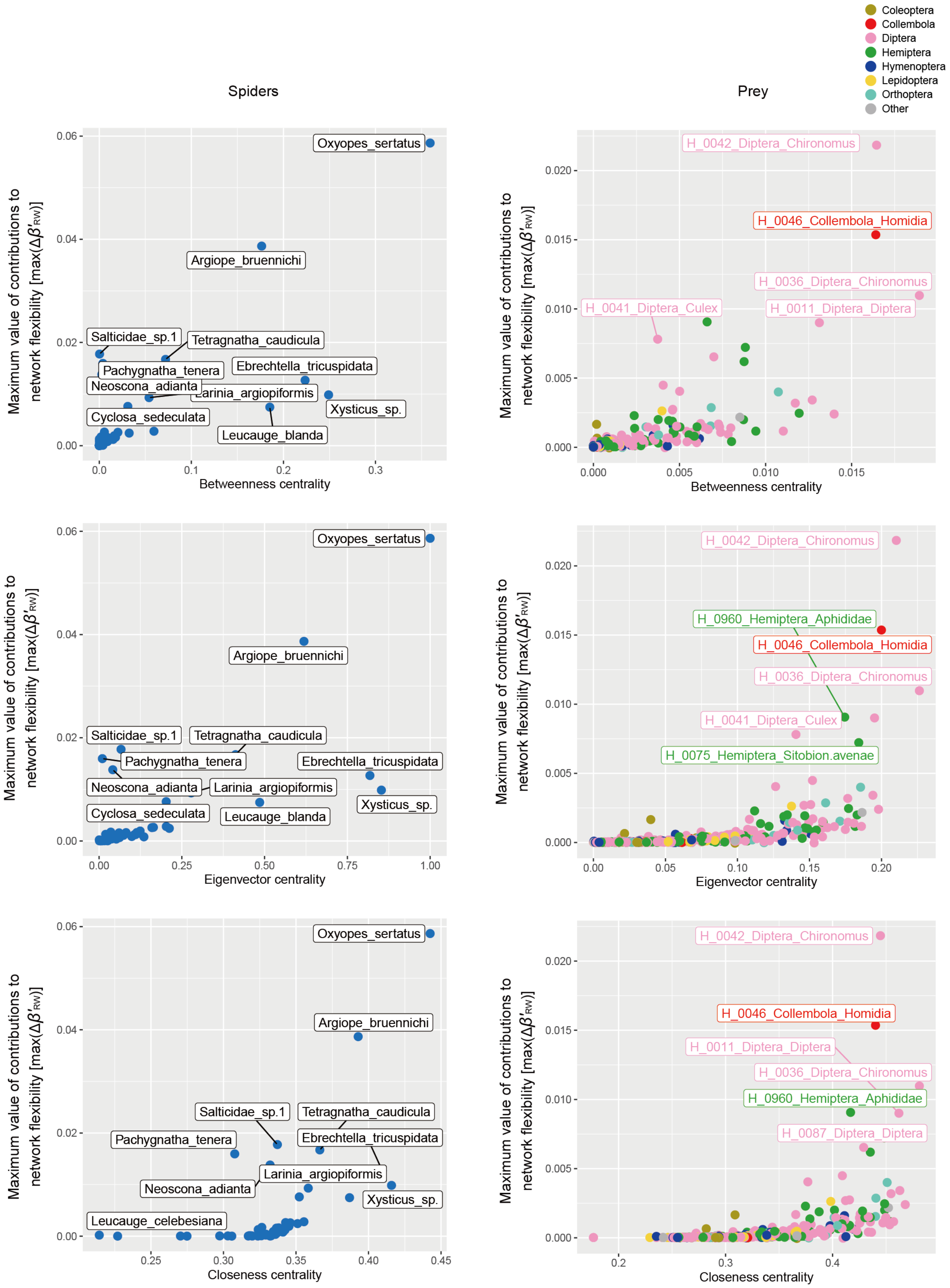

Fig. S4. Network centralities and contributions to network flexibility. The maximum values of contributions to network rewiring effects [$\max(\Delta\boldsymbol{\beta}'_{\mathrm{RW},i})$] (Fig. S3) are plotted against each axis of betweenness, eigenvector, or closeness centrality within the meta-network (Fig. S1). Scores of betweenness were normalized for within each

network so that they varied from 0 (occupation at marginal positions within a network) to 1 (occupation at shortest paths for all pairs of vertices). Likewise, eigenvector centrality was scaled within the range from 0 to 1, while closeness was normalized by dividing raw closeness value by $N - 1$, where N is the number of vertices within the target network.

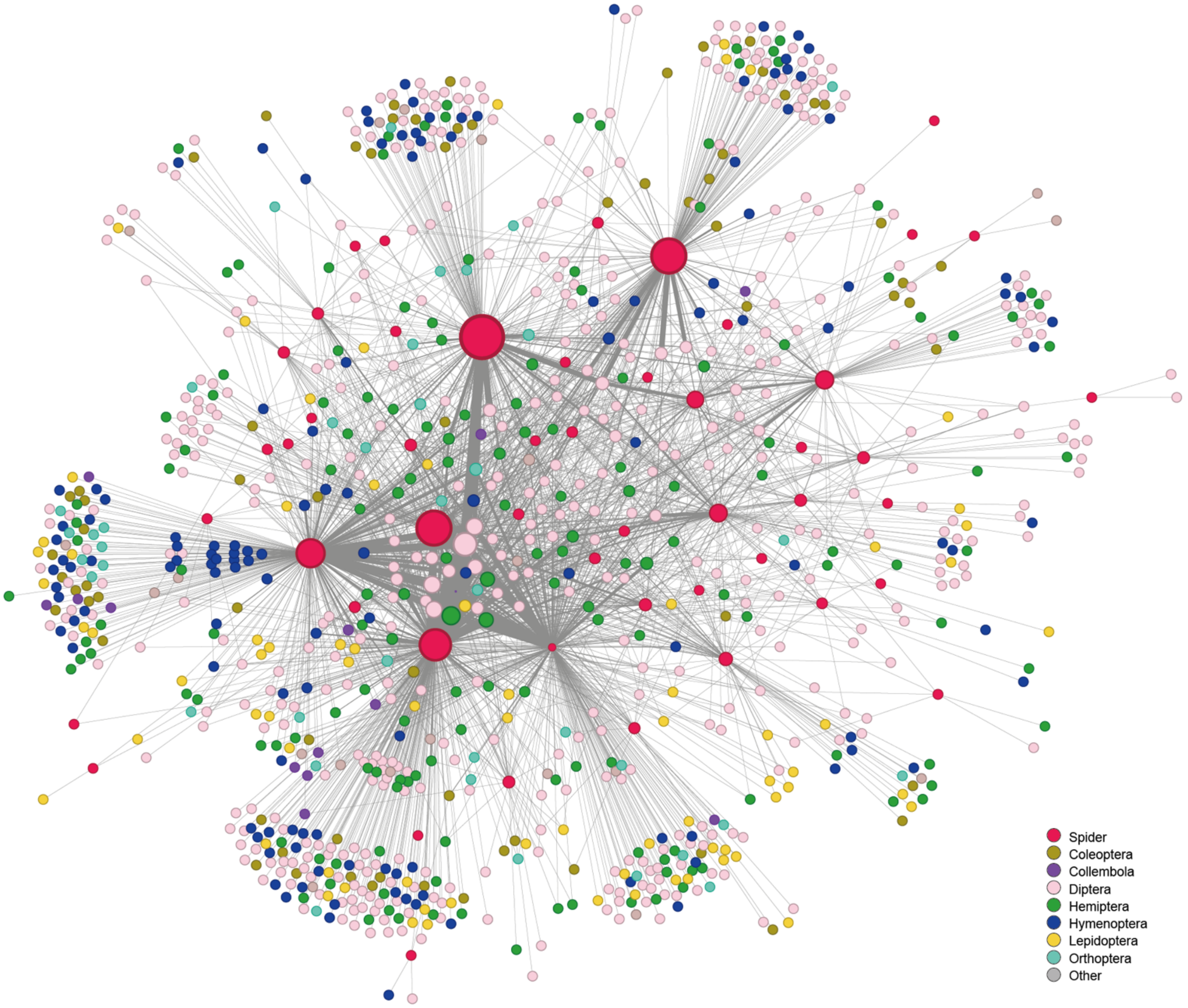

Fig. S5. Species turnover within the meta-network. For each spider species or prey OTU, the maximum values of contributions to dissimilarity in network architecture due to species turnover [max($\Delta\boldsymbol{\beta}'_{\mathbf{ST},i}$)] is represented by vertex (node) size within the meta-network. The thickness of edges (links) indicates prey detection counts. Network ordination was optimized with the ForceAtlas2 algorithm.

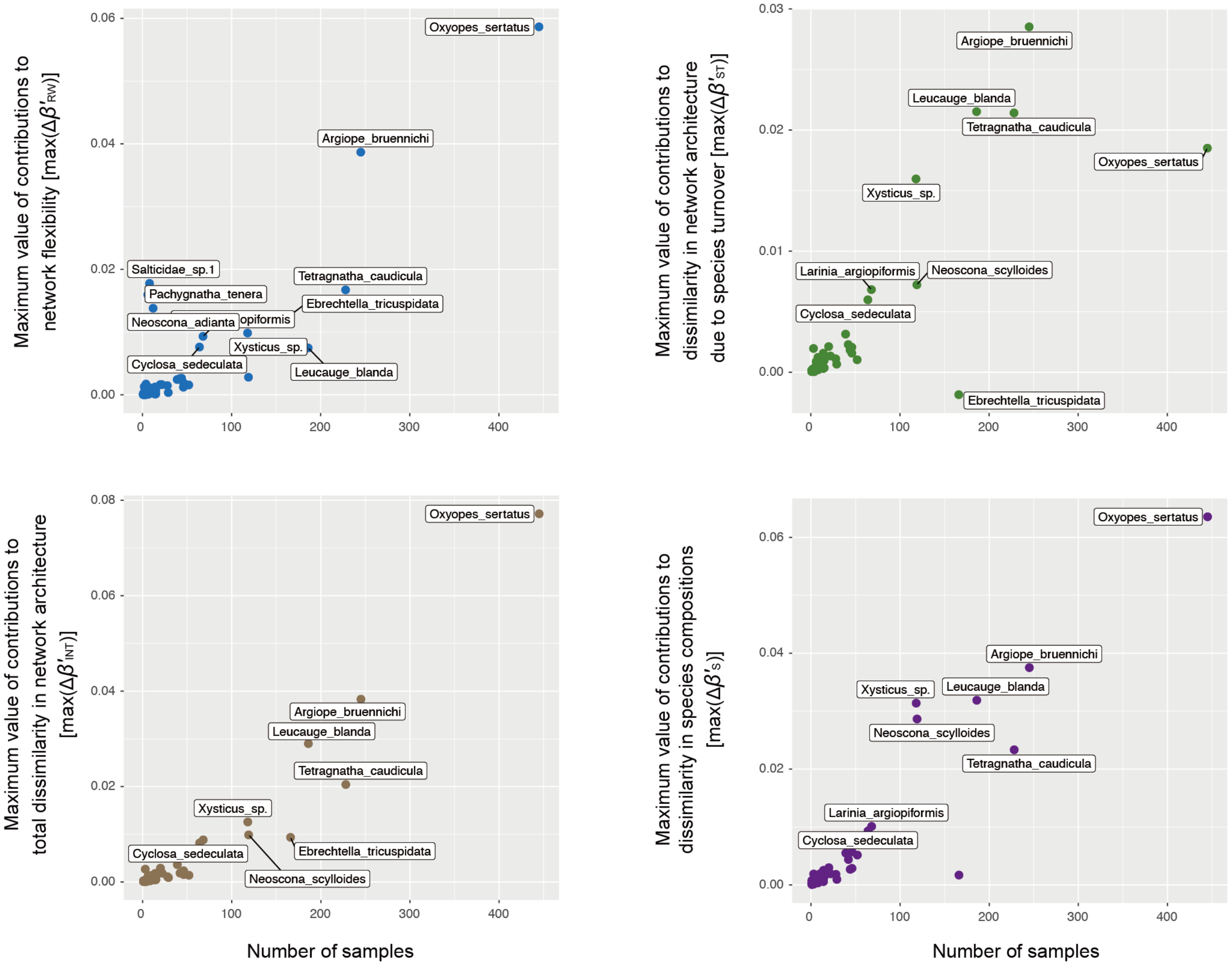

Fig. S6. Number of samples and contribution indices. The maximum values of contributions to network rewiring effects [max($\Delta\boldsymbol{\beta}'_{\mathbf{RW},i}$)], the maximum values of contributions to dissimilarity in network architecture due to species turnover [max($\Delta\boldsymbol{\beta}'_{\mathbf{ST},i}$)], the maximum values of contributions to total dissimilarity in network architecture [max($\Delta\boldsymbol{\beta}'_{\mathbf{INT},i}$)], and the maximum values of contributions to dissimilarity in species compositions [max($\Delta\boldsymbol{\beta}'_{\mathbf{S},i}$)] are plotted against the axis indicating the number of collected spider samples.

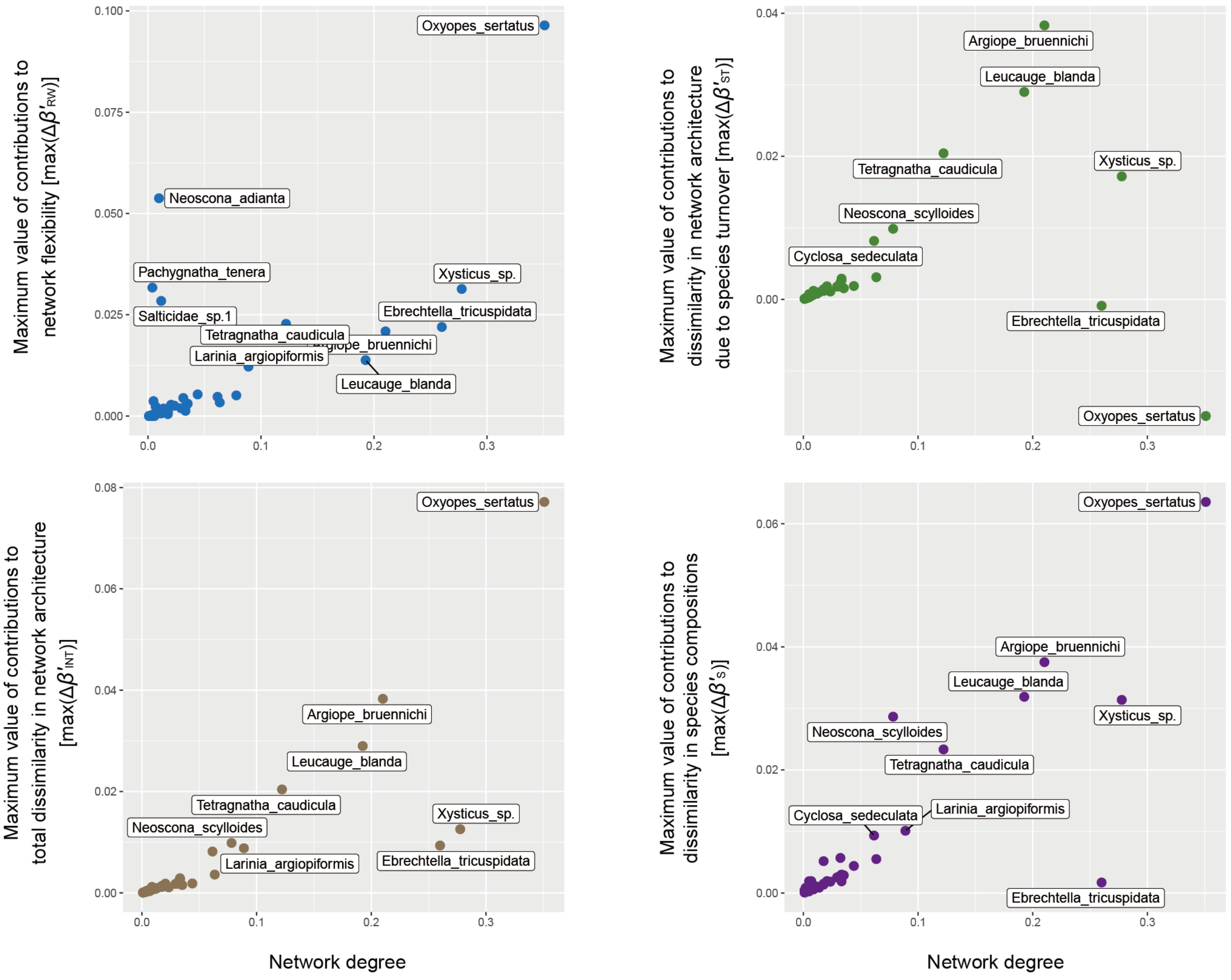

Fig. S7. Contribution indices calculated based on framework 1 (spiders). In the main body of the paper and Figures 2-6, the β-diversity-based indices were calculated based on framework 2, in which common denominators were used in the calculation of $\Delta\boldsymbol{\beta}_{\mathbf{RW}}$ and $\Delta\boldsymbol{\beta}_{\mathbf{ST}}$. For comparison with framework 2, the contribution indices were re-calculated based on framework 1, in which $\boldsymbol{\beta}_{\mathbf{ST}}$ was defined as $\boldsymbol{\beta}_{\mathbf{INT}} - \boldsymbol{\beta}_{\mathbf{RW}}$. The horizontal axis indicates network degree within the meta-network (Fig. S1).

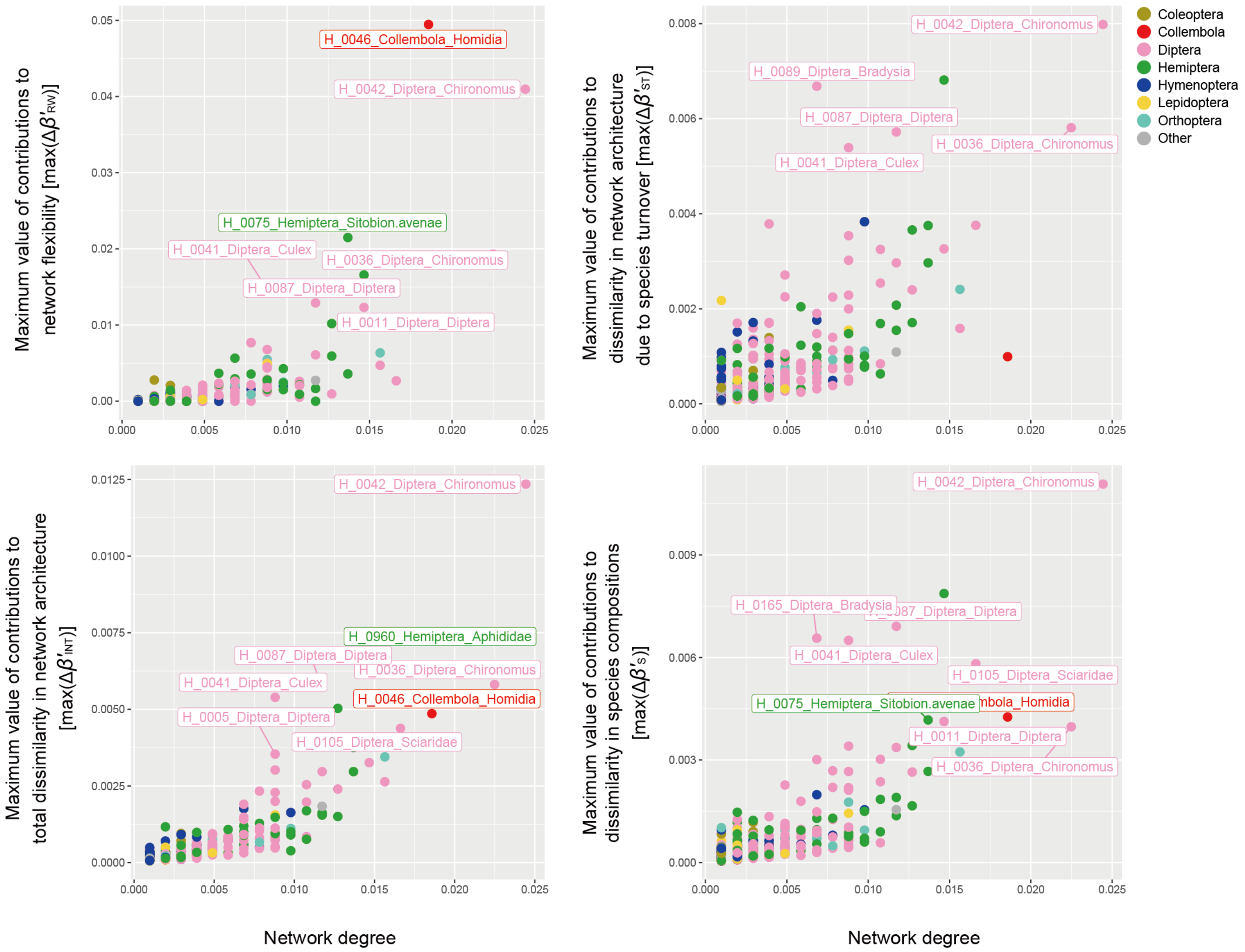

Fig. S8. Contribution indices calculated based on framework 1 (prey). In the main body of the paper and Figures 2-6, the β-diversity-based indices were calculated based on framework 2, in which common denominators were used in the calculation of $\Delta\boldsymbol{\beta}_{\mathbf{RW}}$ and $\Delta\boldsymbol{\beta}_{\mathbf{ST}}$. For comparison with framework 2, the contribution indices were re-calculated based on framework 1, in which $\boldsymbol{\beta}_{\mathbf{ST}}$ was defined as $\boldsymbol{\beta}_{\mathbf{INT}} - \boldsymbol{\beta}_{\mathbf{RW}}$. The horizontal axis indicates network degree within the meta-network (Fig. S1).
